# Left-sided appendicitis managed laparoscopically: a case report

**DOI:** 10.1186/s13256-023-04301-5

**Published:** 2024-01-18

**Authors:** Tamara B. AlKeileh, Sali Elsayed, Raheemah Mahomed Adam, Mozamil Nour, Tarun Bhagchandi

**Affiliations:** 1 Department of General Surgery, Mediclinic Al Noor Hospital, PO BOX 46713, Abu Dhabi, UAE; 2 Department of Radiology, Mediclinic Al Noor Hospital, Abu Dhabi, UAE; 3 Department of General Anesthesiology, Mediclinic Al Noor Hospital, Abu Dhabi, UAE

**Keywords:** Left-sided appendicitis, Laparoscopic surgery, Gut malrotation, Pediatric appendicitis, Abdominal pain

## Abstract

**Background:**

Appendicitis is one of the most common causes of acute abdominal pain and remains the most common abdominal-related emergency seen in emergency room that needs urgent surgery (Yang *et al*. in J Emerg Med 43:980–2, 2012. 10.1016/j.jemermed.2010.11.056, Wickramasinghe *et al*. in World J Surg 45:1999–2008, 2021. 10.1007/s00268-021-06077-5). The characteristic presentation is a vague epigastric or periumbilical discomfort or pain that migrates to the lower right quadrant in 50% of cases. Other related symptoms, such as nausea, anorexia, vomiting, and change in bowel habits, occur in varying percentages. The diagnosis is usually reached through comprehensive history, physical examination, laboratory tests, and radiological investigations as needed. Nowadays, computed tomography of the abdomen and pelvis is considered the modality of choice for definitive assessment of patients being evaluated for possible appendicitis. Anatomical variations or an ectopic appendix are rarely reported or highlighted in literature.

**Case presentation:**

Left-sided appendicitis is a rare (Hu *et al*. in Front Surg 2022. 10.3389/fsurg.2022.896116) and atypical presentation and has rarely been reported. The majority of these cases are associated with congenital midgut malrotation, situs inversus, or an extremely long appendix (Akbulut *et al*. in World J Gastroenterol 16:5598-5602, 2010. 10.3748/wjg.v16.i44.5598). This case is of significance to raise awareness regarding an anatomical variation of the appendix that might delay or mislead diagnosis of appendicitis and to confirm safety of a laparoscopic approach in dealing with a left-sided appendicitis case (Yang *et al*. in J Emerg Med 43:980–2, 2012. 10.1016/j.jemermed.2010.11.056). We report a case of left-sided appendicitis in a 12-year-old child managed successfully via a laparoscopic approach.

**Conclusion:**

Appendicitis remains the most common abdominal-related emergency that needs urgent surgery (Akbulut *et al*. in World J Gastroenterol 16:5598–5602, 2010. 10.3748/wjg.v16.i44.5598). Left-sided appendicitis is a rare (Hu *et al*. in Front Surg 2022. 10.3389/fsurg.2022.896116, Hu et al. in Front Surg 9:896116, 2022. 10.3389/fsurg.2022.896116) and atypical presentation and has rarely been reported. Awareness regarding an anatomical variation of the appendix and diagnostic modalities on a computed tomography scan help avoid delay in diagnosis and management of such a rare entity (Vieira* et al*. in J Coloproctol 39(03):279–287, 2019. 10.1016/j.jcol.2019.04.003). A laparoscopic approach is a safe approach for management of left-sided appendicitis (Yang *et al*. in J Emerg Med 43:980–2, 2012. 10.1016/j.jemermed.2010.11.056, Hu *et al*. in Front Surg 9:896116, 2022. 10.3389/fsurg.2022.896116).

## Background

A 12-year-old boy of Arabic ethnicity, with good school performance, previously medically free and not on any medical treatment, and belonging to a middle class family with no known medical or inherited diseases in his family history, presented to our emergency room accompanied by his parents with typical acute appendicitis symptoms, yet was found on investigation to have an ectopic appendix on the left side of the abdomen.

Familiarity with the anatomy and anatomical variants of the appendix and colon is essential to make the correct diagnosis and choose the appropriate treatment modality without any delay. A majority of cases of patients with left-sided appendicitis have an associated midgut malrotation, situs inversus totalis, or a very long appendix. A computed tomography (CT) scan modality can help in an accurate diagnosis of these cases with anatomical variations and in preoperative planning of surgical approach.

## Introduction

In an acute setting, early diagnosis of acute appendicitis is imperative to prevent further complications [1]. This can be made more difficult with atypical presentations or unusual anatomy of appendix, such as a left-sided appendix. From the surgical approach, laparoscopic intervention is advantageous, as it is minimally invasive with fewer complications; however, the operative technique and duration can be influenced by cases with atypical anatomical variants.

Reviewing medical literature, we only found a few cases that presented left-sided appendicitis or anatomic anomalies of the appendix. Most cases presented were linked to situs inversus. In our abstract, we present a unique case of left-sided appendicitis in a 12-year-old child who was found to have an arrested intestinal rotation that led to the abnormal position of his appendix.

The importance of reporting such cases is to highlight and bring the attention of surgeons to the likelihood of anomalic variations of the appendix, which can lead to a raised level of suspicion and early detection of appendicitis without any delay or complications due to delay.

## Case report

A 12-year-old boy of Arabic ethnicity, with good school performance, previously medically free and not on any medical treatment, and belonging to a middle class family with no known medical or inherited diseases in family history, presented to our emergency room accompanied by his parents complaining of moderate-to-severe abdominal pain in the epigastric region of the abdomen for 1 day prior to presentation to the emergency room.

According to the patient and his mother, on the morning of presentation to our emergency room, the pain migrated and became more prominent in the lower right abdomen. He also developed recurrent vomiting and diarrhea episodes from the start of his illness episode, 1 day prior to his presentation to our emergency room, along with anorexia and feeling of fatigue; no fever was documented, but he had episodes of chills. On inquiring from mother, the patient had no recent history of upper respiratory tract infection and had no previous medical conditions or surgical interventions.

On physical examination, the patient was conscious, alert, and oriented, with no neurological deficit; he seemed ill and slightly dehydrated (with dry eyes and skin), and he was in pain. His vital signs showed a low-grade fever of 37.2 °C, pulse rate of 95 beats per minute, and blood pressure of 100/70 mmHg. His weight was 43 kg, and height was 152 cm.

On throat examination, he had no throat congestion nor enlarged tonsils. On chest examination, he had resonant chest, equal air entry at both lungs, and no wheezes or crackles. On abdominal physical examination, he was found to have generalized abdominal guarding and lower abdominal tenderness, with the most prominent tenderness in the lower right abdomen, and positive rebound tenderness in right iliac fossa. Other signs of appendicitis, such as Rovsing’s, psoas, and obturator signs, were not elicited, as the patient was in severe pain and withdrawing from any physical examination. A digital rectal examination was not performed, as the patient did not have constipation or blood in his stool, hence, in view of the clinical scenario, a digital rectal examination was thought to not add additional value in diagnosis.

Keeping in mind possible differential diagnosis of patient presentation in this case, gastroenteritis, mesenteric lymphadenitis, Meckel’s diverticulum, pyelonephritis or nephrolithiasis, typhilitis, testicular torsion, and acute appendicitis remain on the top of the list to prove or rule out, hence, further investigations were done accordingly [2].

The laboratory results were significant for leukocytosis (12.14 × 10^9^ cells/L) with neutrophilia (80.4%). His hemoglobin was 12.4 g/dL, and CRP was only 3.0 mg/dL; his blood urea nitrogen/creatinine and electrolytes were normal.

An abdominal ultrasound revealed enlarged mesenteric lymph nodes, but the appendix could not be visualized due to bowel gases.

A CT scan of the abdomen and pelvis was performed, which showed an inflamed and thickened appendix measuring 1.1 cm in diameter, with mild free fluid in the pouch of Douglas. The findings were suggestive of acute appendicitis changes. The appendix was localized retrocecal, but the cecum was at the midline, and the tip of the appendix was seen at the left side of the urinary bladder.

**Fig. 1:**
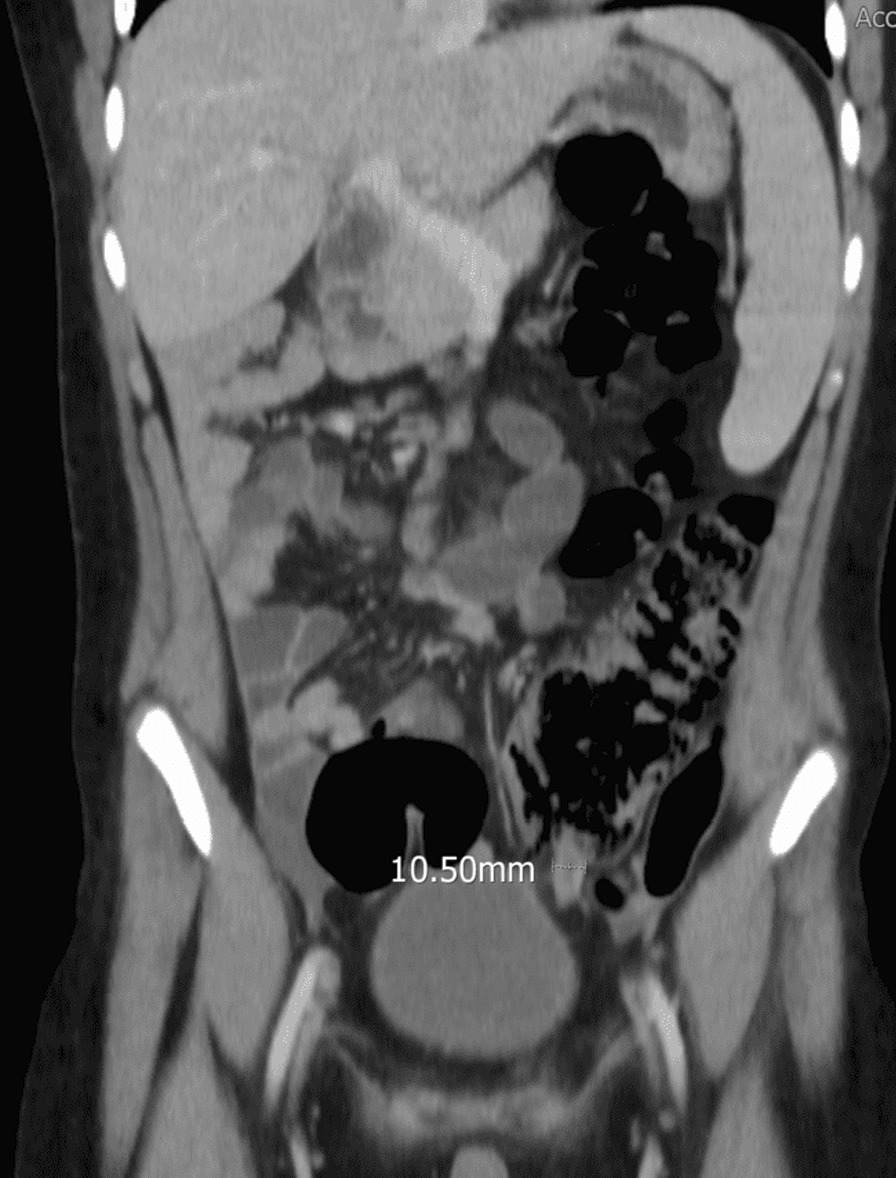
CT of the abdomen showing enlarged appendix (10.50 mm) located to the left of the urinary bladder

**Fig. 2:**
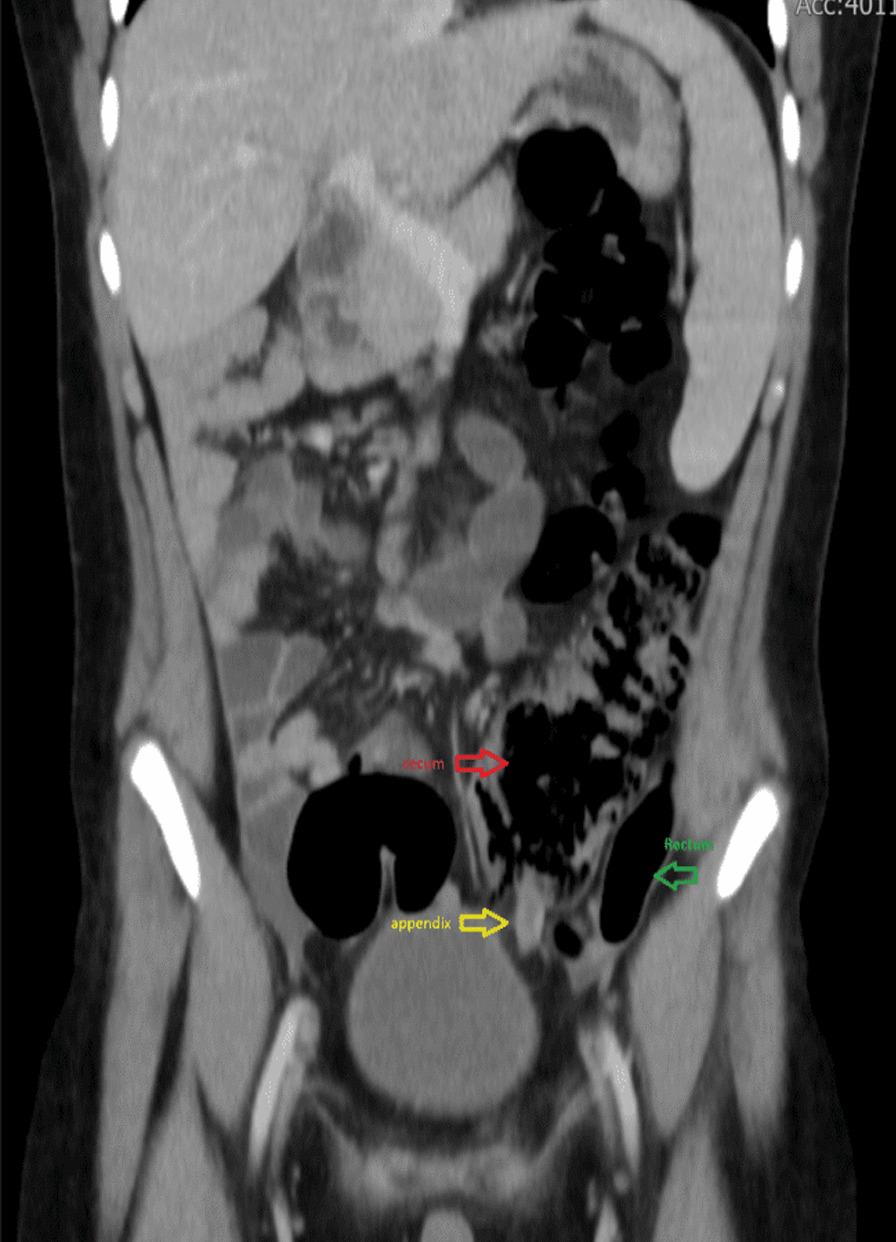
CT of the abdomen (coronal view) showing the appendix (yellow) between the cecum (red) and rectum (green)

**Fig. 3:**
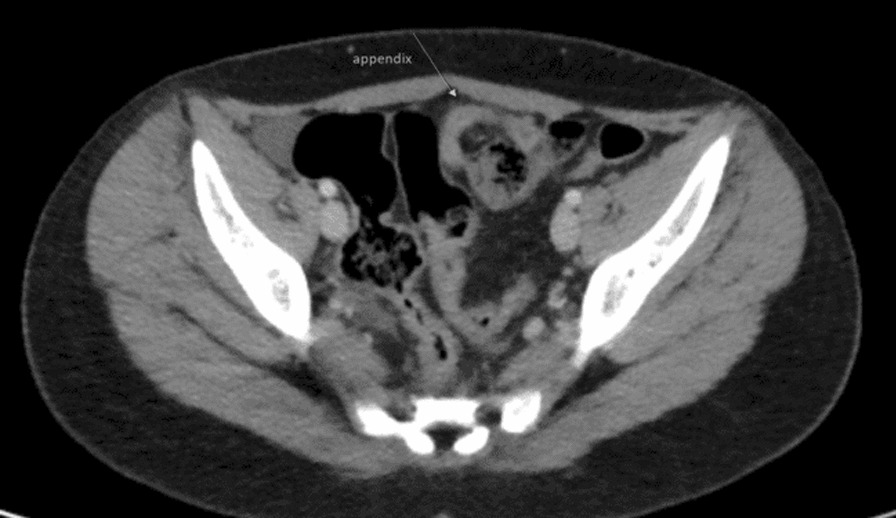
CT of the abdomen (axial cut) showing location of appendix toward the left side of the abdomen

**Fig. 4:**
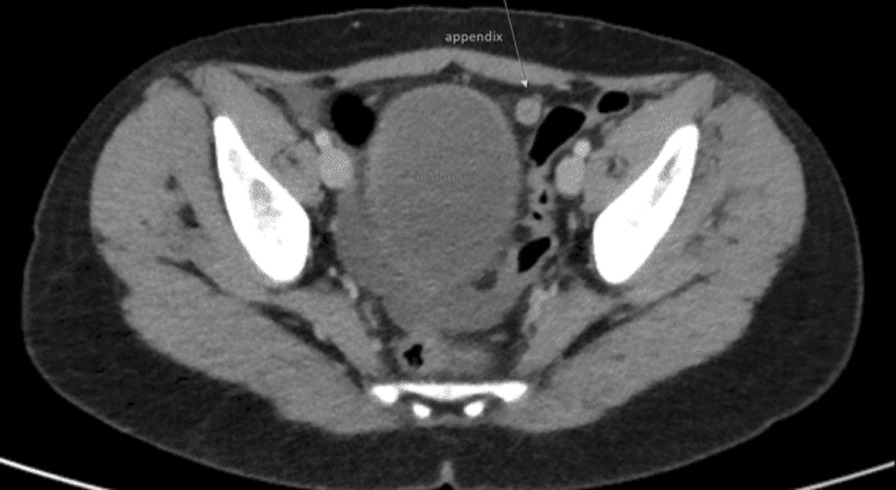
CT of the abdomen (axial cut) illustrating the appendix (arrow) located laterally to the left of the urinary bladder

Enlarged reactive mesenteric lymph nodes (Figs. 1, 2, 3, 4).

The patient was admitted to the hospital and was started on intravenous (IV) fluids and intravenous antibiotics; he received ceftriaxone 1 g IV every 12 h, metronidazole 500 mg IV every 8 h, and was prepared for surgery.

**Fig. 5:**
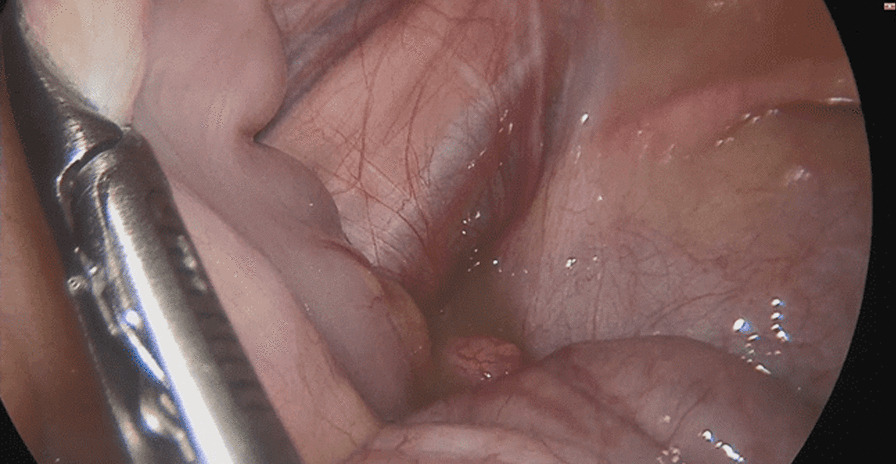
Laparoscopic view showing free pus in the lower left abdomen at the site of the inflamed appendix

**Fig. 6:**
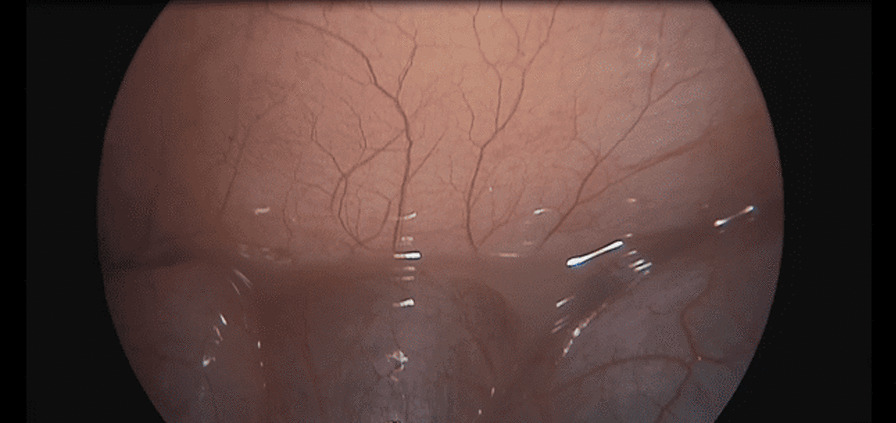
Laparoscopic view showing free pus between the small bowel loops on the right of the abdomen

**Fig. 7:**
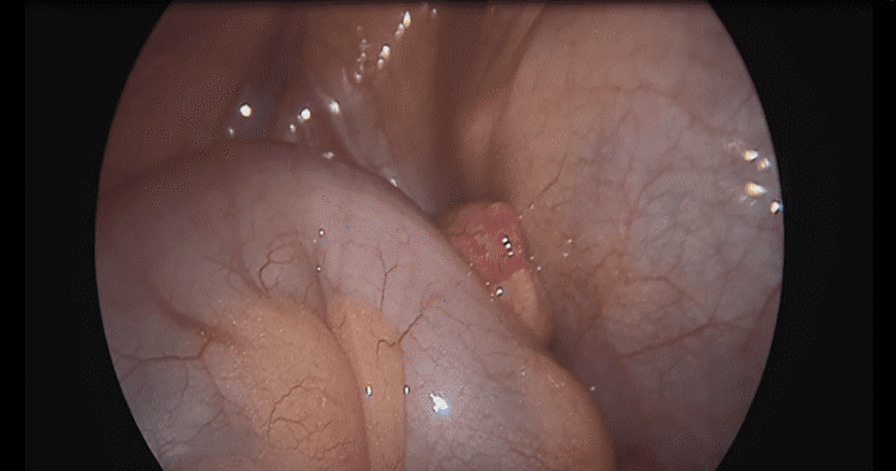
Laparoscopic view showing the appendix tip can be seen at the left iliac fossa between the cecum and sigmoid

**Fig. 8:**
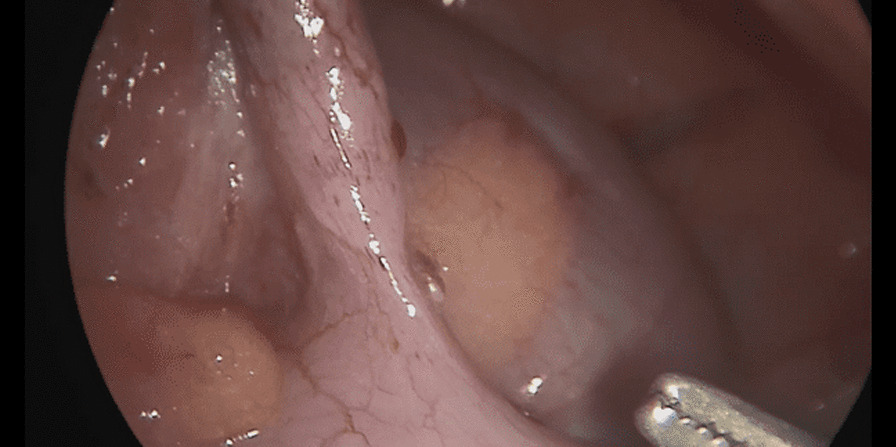
Laparoscopic view showing the appendix base arising from the cecum seen at the left iliac fossa with the sigmoid colon behind

**Fig. 9:**
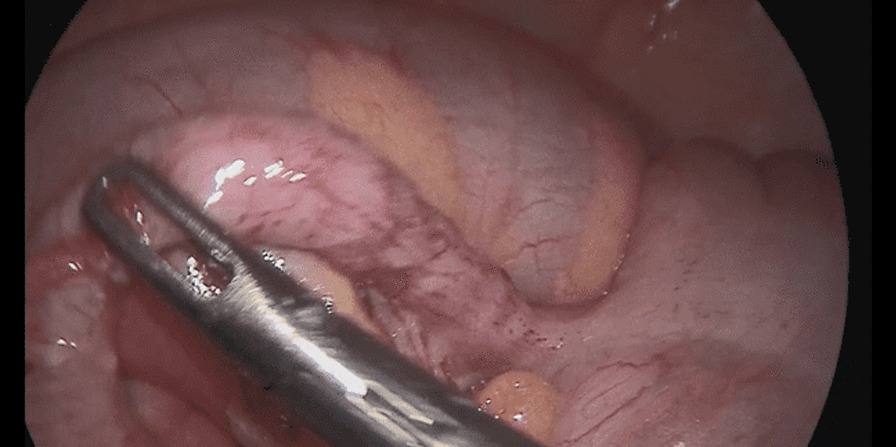
Laparoscopic view showing the left-sided appendix with the cecum and sigmoid seen in the lower left abdomen

**Fig. 10:**
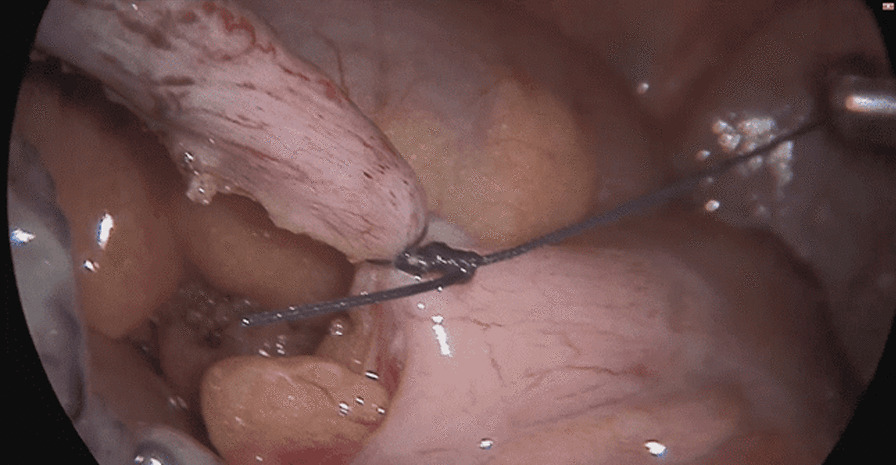
Laparoscopic view showing the left appendix ligation of base

The patient underwent a laparoscopic exploration, and the surgical findings showed free pus collections at the pelvis, the right paracolic gutter, around the liver, and in between bowel loops/generalized peritonitis. The cecum and ascending colon were found to be pushed to left abdomen, adjacent to the descending and sigmoid colon, and the appendix tip was found to be in the left side of the pelvis to the left of the bladder and was inflamed, especially distally at the tip, measuring up to 1.2 cm in diameter, approximately, with a healthy-looking base arising from the cecum. (Figs. 5, 6, 7, 8, 9, 10).

The trocars were placed as follows: a infraumbilical 10 mm trocar for camera port and a 5 mm trocar inserted through the incision at suprapubic area and another 10 mm trocar inserted through the incision at the right iliac fossa (in a regular right-sided appendix, this port is usually placed at the left iliac fossa); the monitor was placed on the left side of the patient and the surgeon and assistant on the right side.

The appendix was dissected and ligated at the base using an endoloop and then removed in an endobag. A pus swab culture was taken, and suctioning of the pus and abdominal lavage was performed, leaving a drain at the pelvis and another drain at the subhepatic and right paracolic area, where pus collections were found.

Postsurgery, the patient recovered well; he passed flatus on the second postoperative day and tolerated an oral soft diet. The drains were removed on the third postoperative day, and the patient was discharged home in good condition on the fourth postoperative day.

The histopathological result of the removed appendix revealed acute suppurative appendicitis and periappendicitis, which was negative for malignancy, and the pus culture had no growth.

Postdischarge from the hospital, the patient returned to the clinic accompanied by his parents after 1 week and was doing well clinically with no complaint. No wound infection developed. No further visits to the hospital or clinic related to his surgical condition were required since that date.

## Discussion

In summary, the case we presented is a case of 12-year-old, previously healthy, boy not known to have any medical illnesses who presented with symptoms of abdominal pain, recurrent vomiting, episodes of diarrhea, fatigue, anorexia, and chills. The boy looked ill and had lower abdominal tenderness with positive rebound tenderness on the lower right quadrant, yet investigations showed that he had an ectopic inflamed appendix on the left side, evident on an abdominal CT scan, which helped the surgical teams to plan his surgery accordingly. He underwent a successful laparoscopic appendectomy, and intraoperative findings showed that all the large bowel was on the left side of the abdomen, while the small bowel was on the right side of abdomen. In addition to the severely inflamed left-sided appendix, he was also found to have generalized peritonitis with multiple free pus collections all over the abdomen. He recovered well postoperatively and was discharged in good condition on the fourth day, and he was doing very well in his follow-up at the clinic.

The unique aspect of this case was the absence of situs inversus, and that the preoperative diagnosis led the surgical planning to successfully help and safely manage this case laparoscopically.

Left-sided appendicitis is a rare condition and, therefore, easy to misdiagnose, which could be challenging for surgeons to handle. A delay in diagnosis can lead to serious complications, such as a periappendiceal abscess, perforation, or gangrene [1–3].

Familiarity with the anatomy and anatomical variants of the appendix and colon is essential to make the correct diagnosis and choose the appropriate treatment modality without any delay [3]. Hence, a discussion on the anatomical variations and congenital abnormalities of these structures is encouraged to help in keeping medical and surgical teams alert to different possibilities and to avoid missing a patient’s condition.

A majority of cases of patients with left-sided appendicitis have associated midgut malrotation, situs inversus totalis, or a very long appendix [4]. Intestinal malrotation is a congenital rotational anomaly that occurs as a result of an arrest of normal rotation of the embryonic gut, and is said to occur in 1 in 6000 live births [5].

In our patient’s case, there was no situs inversus, despite the cecum and ascending colon being on the left side of the abdominal cavity, as well as the descending colon and sigmoid. The small intestine bowel loops were located on the right side of the abdomen, and the appendix was lying down in the pelvis to the left side of the bladder, far away from the lower right quadrant, which represents a sort of intestinal malrotation or arrested rotation.

Additionally, what is interesting in this case is the presence of typical appendicitis signs and symptoms in terms of pain migration, from the epigastric region to the lower right abdomen, and the most prominent tenderness being at the right iliac fossa, despite the later-identified location of the appendix on left side. This symptom also appears in about 18–34% of patients with situs inverses and midgut malrotation [4]

Keeping in mind possible differential diagnosis of patient presentation in this case, as gastroenteritis, mesenteric lymphadenitis, Meckel’s diverticulum, pyelonephritis or nephrolithiasis, typhilitis, and acute appendicitis remained on the top of the list of our differential diagnosis to prove or rule out, further investigations were done accordingly and proved the diagnosis of acute appendicitis with an ectopic appendix on the left.

In our patient’s case, the findings of right iliac fossa tenderness and rebound tenderness could additionally be attributed to the presence of free fluid collection in the right paracolic gutter, found intraoperatively as part of generalized peritonitis associated with the inflammation of his appendix. Another hypothesis is that it is assumed that, even though the viscera are transposed, the nervous system may not show the corresponding transposition, which may result in confusing signs and symptoms. In about 18.4–31% of patients with situs inversus totalis (SIT) and midgut malrotation (MM), the pain caused by left-sided appendicitis was reported in the lower right quadrant [4].

Of course, another interesting aspect of this case is the overwhelming inflammatory response to appendicitis despite the short clinical history the patient presented with.

Whether this can be attributed to an under developed omentum, which usually is the case in younger children, or to delay of presentation, the abnormal anatomy of the bowel and ectopic appendix, which led to progression of disease without obvious symptoms until the patient developed severe abdominal pain and presented to the emergency room, could not be determined.

Here, we can appreciate the benefit of an abdominal CT scan in such controversial cases where clinical signs and symptoms, as well as laboratory tests showing the presence of mild leukocytosis and neutrophilia, are correlated with the diagnosis of appendicitis, but the ultrasound scan failed to detect the appendix. It is known that ultrasound is the preferred initial examination in pediatric cases [6], because it is nearly as accurate as CT scans for the diagnosis of acute appendicitis in this population without the use of ionizing radiation [7].

However, in this case, the ultrasound modality missed the findings, and the CT scan proved the high clinical suspicion of acute appendicitis and added the detection of an ectopic appendix. This was necessary in aiding preoperative planning, reducing operative time and complications, and guiding the surgeon to the exact location of the inflamed appendix.

Contrast-enhanced, thin-section (0.5 mm) CT scanning has become the preferred imaging technique in the diagnosis of acute appendicitis and its complications, with a high diagnostic accuracy of 95–98% [6, 8, 9].

In case abdominal CT is not available or could not be done in such a case scenario, then laparoscopic exploration is advised to avoid missing or delaying the diagnosis, which might end up causing serious harm to the patient’s health. Having a more refined understanding of the patients’ anatomy preoperatively is a necessary factor in deciding equipment needed, placing the port, and avoiding accidental injury during exploration, especially in rare anatomical cases. Once ectopic appendicitis is diagnosed, surgical treatment can be done laparoscopically with a high degree of safety [1, 5].

Laparoscopic appendectomy is a safe and effective method for the treatment of ectopic appendicitis [1, 5, 10]. Laparoscopically, the abdominal and pelvic cavity can be explored to determine the diagnosis of ectopic appendicitis and exclude acute abdomen caused by other diseases. The laparoscopic approach is minimally invasive and reduces the incidence of wound infection and hernia, and it offers faster postoperative recovery and fewer complications [11].

Therefore, it is a safe and effective method for the treatment of ectopic appendicitis and the preferred modality of choice, provided the surgeon has good laparoscopic experience.

The question that arises in this case, and other cases with malrotation, for further discussion is if there is any need for further intervention for the malrotation in absence of any significant symptoms or complications related to it.

## Conclusion

Left-sided appendicitis is a rare and atypical presentation and has been rarely reported. The majority of these cases are associated with congenital midgut malrotation, situs inversus, or an extremely long appendix [4].

Familiarity with the anatomy and anatomical variants of the appendix and colon is essential to make the right diagnosis and choose the right treatment modality with no delay.

Diagnosis is usually reached through a comprehensive history, physical examination, laboratory tests, and radiological investigations as needed.

Nowadays, CT of the abdomen and pelvis is considered the modality of choice for a definitive assessment of patients being evaluated for possible appendicitis [8].

Once ectopic appendicitis is diagnosed, a laparoscopic appendectomy can be done safely.

The laparoscopic approach reduces the incidence of wound infection and hernia and offers faster postoperative recovery and fewer complications [1, 5, 11].

## Data Availability

All documents related to this case are available on request.
